# Harnessing the Microbiome: A Comprehensive Review on Advancing Therapeutic Strategies for Rheumatic Diseases

**DOI:** 10.7759/cureus.50964

**Published:** 2023-12-22

**Authors:** Priyadarshini Bhattacharjee, Karim Arif Karim, Zahid Khan

**Affiliations:** 1 Acute Medicine, Cambridge University Hospital NHS Foundation Trust, Cambridge, GBR; 2 School of Clinical Medicine, University of Cambridge, Cambridge, GBR; 3 Medicine and Surgery, Kamuzu University of Health Sciences, Blantyre, MWI; 4 Acute Medicine, Mid and South Essex NHS Foundation Trust, Southend-on-Sea, GBR; 5 Cardiology, Bart’s Heart Centre, London, GBR; 6 Cardiology and General Medicine, Barking, Havering and Redbridge University Hospitals NHS Trust, London, GBR; 7 Cardiology, Royal Free Hospital, London, GBR

**Keywords:** autoimmune connective tissue disease, future therapeutic strategies, intestinal microbiome, human microbiome, autoimmune rheumatic diseases

## Abstract

Rheumatic diseases are a group of disorders that affect the joints, muscles, and bones. These diseases, such as rheumatoid arthritis, lupus, and psoriatic arthritis, can cause pain, stiffness, and swelling, leading to reduced mobility and disability. Recent studies have identified the microbiome, the diverse community of microorganisms that live in and on the human body, as a potential factor in the development and progression of rheumatic diseases. Harnessing the microbiome offers a promising new avenue for developing therapeutic strategies for these debilitating conditions. There is growing interest in the role of oral and gut microbiomes in the management of rheumatoid arthritis and other autoimmune disease. Microbial metabolites have immunomodulatory properties that could be exploited for rheumatic disorders. A wide range of microorganisms are present in the oral cavity and are found to be vulnerable to the effects of the environment. The physiology and ecology of the microbiota become intimately connected with those of the host, and they critically influence the promotion of health or progression toward disease. This article aims to provide a comprehensive overview of the current state of knowledge on oral and gut microbiome and its potential future role in the management of rheumatic diseases. This article will also discuss newer treatment strategies such as bioinformatic analyses and fecal transplantation.

## Introduction and background

The microbiome is an ecosystem of microorganisms that inhabit the human body and is composed of bacteria, fungi, and viruses [1-3]. This microbial ecosystem is closely related to human health and the development of autoimmune diseases, including rheumatic diseases [1,4]. The intestinal microbiome is of particular interest as it is affected by multiple factors such as geographical location, diet, sex, and age [4]. These factors can be modified through treatments with probiotics, prebiotics, and other microbiome-altering therapies, which could potentially modulate the composition of the intestinal microbiome [4]. Studies have shown that patients with rheumatoid arthritis and type 1 diabetes have a unique intestinal microbiome profile different from that of healthy individuals [4]. Furthermore, this differential composition impacts the immune system [4], and the mechanisms behind this effect are varied [1]. Specific bacterial communities in the mouth, lung, and skin have been associated with the pathogenesis of rheumatic diseases, and the microbiota has been either correlated or causally related to the development of rheumatoid diseases [1]. Animal models have also established the biological connection between microbiota and the development of synovitis, and germ-free (GF) conditions can prevent the development of arthritis [5]. The microbiota has been shown to induce T helper 17 (Th17) cells and secretory immunoglobulin A (IgA) responses to segmented filamentous bacteria [1] and interact with rheumatic diseases apart from the local mucosal immune reaction [4]. Artificial intelligence approaches such as machine learning can be applied to microbiome biomarkers research in rheumatic diseases, which could lead to the improvement of patient profiling and treatment personalization [2]. Thus, the microbiome offers unique opportunities to better understand the aspects of pathogenesis in rheumatic diseases [2], making it an attractive area of research for rheumatologists, immunologists, and microbiologists [4].

There is not much recent evidence on the pros and cons of the recent advancements in the therapeutics of rheumatic disease. The purpose of this review article was to explore the advantages and disadvantages of gut microbiota on rheumatism, identify the potential knowledge gap, and make recommendations for future studies in this regard. We performed a thorough literature search from 1st March 2023 to 1st May 2023 on various search engines including PubMed, Google Scholar, and EMBASE. The search terms were “advanced therapeutic strategy,” “rheumatic disease,” “gut,” and “microbiome,” and the Booleans used were AND, OR, and NOT. Only full-text articles in English were used. Case reports and case series were excluded.

## Review

Importance of considering the microbiome in therapeutic strategies for rheumatic diseases

Considering the microbiome in therapeutic strategies for rheumatic diseases is essential due to its potential role in rheumatoid arthritis (RA) pathogenesis. Studies have observed a correlation between the gut microbiome and RA, but not necessarily causation [5]. The mucosal sites exposed to bacterial antigens may lead to tolerance breaks in rheumatic diseases, and the gut may represent the initial site of tolerance breaks [3]. Additionally, artificial intelligence (AI) can be used to study the relationship between oral microbiome and rheumatic diseases [2]. Prediction of the link between oral microbiome and rheumatic diseases is an important area of research [2]. Previous studies have reported that the subgingival microbiota differs significantly between RA and healthy individuals and that *Porphyromonas gingivalis* on the total tongue biofilm is significantly associated with RA disease activity [2]. Moreover, *Cryptobacterium curtum* is increased in RA patients, and *Aggregatibacter actinomycetemcomitans* is proposed to connect periodontitis to RA for its ability to induce citrullinated autoantigens [2]. Furthermore, LtxA-induced protein citrullination may play a role in anti-citrullinated protein antibody (ACPA)abt production in genetically susceptible subjects [2]. Microbial metabolites, such as short-chain fatty acids (SCFAs) and medium-chain fatty acids (MCFAs), have immunomodulatory properties that could be exploited for rheumatic disorders [3]. Furthermore, dysbiosis can be partially resolved with disease-modifying antirheumatic drugs (DMARDs), and the microbiome can predict response to DMARDs [2]. Finally, the effect on the oral microbiome is greater than that on the gut microbiome [2], and further validation through human studies and mechanistic approaches in gnotobiotic mice is required to evaluate the immune events in response to microbial perturbation in RA [5], making consideration of the microbiome in therapeutic strategies for rheumatic diseases important [2].

Benefits of harnessing the microbiome in the treatment of rheumatic diseases

Recent research has uncovered the benefits of harnessing the microbiome in the treatment of rheumatic diseases, such as RA and spondylarthritis [6]. Unsurprisingly, diet has been established as a major factor in influencing the composition of the intestinal microbiota, which is related to the development and progression of rheumatic diseases [6]. For instance, probiotic bacteria can help to correct gut dysbiosis and promote healthy microbiota [6]. Probiotic supplementation has been found to increase antioxidant defenses and improve oxidative/nitrosative profiles in RA patients as well as improve disease activity and inflammatory status when combined with conventional medication [6]. Additionally, enrichment of certain bacterial families, such as *Prevotellaceae*, has been connected to RA onset, and dysbiosis has been observed in patients before the onset, or at diagnosis, of RA [7]. Fortunately, treatment has been known to partially resolve the dysbiosis associated with RA [7]. In addition, beverages, such as coffee, tea, and wine, have been shown to possess the potential to alter the microbiome in the context of RA [7]. The contents of these beverages can interfere with immune signaling pathways beneficial for RA, which can have downstream effects on inflammatory pathways related to RA [7]. As such, a multidisciplinary team should be able to leverage the growing body of evidence on beverages and dietary patterns for patient management in RA [7]. Furthermore, the administration of probiotic bacteria may have a beneficial effect on the inflammatory activity of RA, in addition to the improvement of intestinal barrier function [6]. Probiotics may also have a positive synergistic effect with DMARDs in treating RA as well as regulating cytokine production in RA patients [6]. Other medications with antibiotic properties, such as hydroxychloroquine and tetracyclines, have been included in the therapeutic armamentarium of RA [5]. Sulfasalazine, a drug that combines a sulfa antibiotic with a salicylate, is a DMARD approved by the FDA for the treatment of RA, IBD, and AS [5]. Harnessing the microbiome and altering the composition of intestinal bacteria can lead to therapeutic regimens for inflammatory arthritis while understanding the immune response to gut microbiota in rheumatic diseases is an additional benefit of this strategy [5]. For example, *Lactobacillus bifidus*-monocolonized Il1rn−/− mice have been found to develop a high incidence of severe joint disease in a Th17- and TLR4-dependent manner [5]. Therapies targeting intestinal bacteria have been part of the antirheumatic strategy for many decades, and the notion that certain classes of antibiotics target microorganisms based on Gram-stain properties has become outdated [5]. Moreover, indiscriminate use of antibiotics at an early age could potentially determine the fate of various conditions, including obesity and even autoimmune disorders [5].

Current strategies to harness the microbiome in the treatment of rheumatic diseases

The current strategies to unlock the potential of the microbiome in the treatment of rheumatic diseases is a complex topic, which requires further study to move from correlation to causation. This requires understanding the existing knowledge and how to navigate the future directions of research [8,9]. The proposed strategies include leveraging the symbiotic microbes to reduce or control the disease, molecular studies, gnotobiotic mouse models, and metagenomic studies [10-12]. The shifts in the diet, such as high fat and high carbohydrate, have been associated with changes in the gut microbiota [13]. Furthermore, the drug-microbiome interface is a key factor in understanding how to manage the disorders [14]. At the same time, the challenge is to link the acquired knowledge of microbiomes to translational forest management [15]. Additionally, the immunological aspects of the microbiota along with innovative treatments that may safely harness the microbiota are being considered [16-18]. Thus, it is clear that, though effort is being made, there are still potential limitations and consequences of the current strategies which must be considered.

Advantages and disadvantages of current strategies

Current strategies are designed to identify the underlying factors and associations of immune-mediated rheumatic diseases. Bioinformatic analyses are used to classify and quantify microbial composition [19]. The goal is to create a robust database of patient data that can aid in disease identification [19]. These strategies also help to overcome the limitations in characterizing and quantifying microbiota composition [19]. However, these strategies also have certain drawbacks [20]. For example, there are key knowledge gaps that must be filled to maximize microbial functions and improve crop performance [20]. Additionally, there are limitations to managing agricultural microbiomes [20]. Despite these drawbacks, the efforts to characterize microbial communities have illuminated the complex interacting domains of crop-associated microbiomes that contribute to agroecosystem health [20].

Strategies used to manipulate the microbiome to treat rheumatic diseases

Strategies for Microbiome Manipulation in Rheumatic Diseases

Innovative strategies like fecal microbiota transplantation, probiotics, and orthogonal niche engineering are actively being employed to target the microbiome and potentially treat rheumatic diseases [18,20]. The aim is to restore the delicate balance between the microbiome and the host, offering a new frontier in therapeutic approaches [18]. However, these strategies face challenges and opportunities inherent in microbiome research [9]. Despite landmark studies in autoimmunity and rheumatology, the clinical impact has been limited, primarily due to the complexity of controlling the environment in microbiome studies [9]. Advances in related fields, including the use of animal models and the development of new technologies, are being explored to address these challenges and pave the way for effective microbiome-targeted treatments [9].

Potential Therapeutic Strategies for Rheumatic Diseases

Microbiome research presents a myriad of opportunities for personalized therapeutic strategies in rheumatic diseases, offering diverse approaches to target the microbiome [2,3,21]. These potential strategies include antibiotic therapy, prebiotics, probiotics, fecal microbiota transplantation (FMT), dietary interventions, manipulation of oral microbiota, and specific microbiota-targeted therapies. For example, dietary interventions, like the Mediterranean diet, have demonstrated positive impacts on gut microbiota, suggesting a potential avenue for therapeutic benefits [3]. Additionally, targeting microbial and dietary metabolites, as well as specific bacterial species, holds promise in addressing rheumatic disorders [3,5,22]. The bidirectional crosstalk between the microbiota and hosts opens avenues for therapeutic interventions, including the use of disease-modifying antirheumatic drugs [2].

Advantages and Disadvantages of Potential Therapeutic Strategies

Promising potential therapeutic strategies include maintenance therapy with traditional agents like methotrexate (MTX), which has demonstrated effectiveness and the ability to induce a remission-like state [23]. Conventional and biologic DMARDs show success in treating patients, although their effectiveness may vary [24]. Exploring topical jakinibs (also known as Janus kinase [JAK] inhibitors) as a potential therapeutic strategy, with their ability to preserve therapeutic efficacy and potentially mitigate adverse effects resulting from systemic JAK inhibition, is a notable advancement [24]. Symptomatic treatment with non-steroidal anti-inflammatory drugs (NSAIDs) may not interfere with underlying immune-inflammatory events or retard joint destruction, emphasizing the importance of disease-modifying strategies [23]. However, further research is essential to comprehensively evaluate the effectiveness and potential disadvantages of these therapeutic strategies in treating rheumatic diseases [23].

Potential therapeutic strategies to target the microbiome in rheumatic diseases

Restoring Microbial Diversity and Composition

Recent research highlights the potential of therapeutic strategies to target the microbiome in rheumatic diseases by focusing on restoring microbial diversity, abundance, and composition [25]. This approach recognizes the intricate relationship between the gut microbiome and rheumatic diseases. Studies, such as one involving the *Bifidobacterium longum* RAPO strain, showcase promising avenues for inhibiting key mediators like interleukin-17 (IL-17) associated with autoimmune diseases like RA [26]. Notably, alterations in specific bacterial taxa, such as *Bifidobacterium*, *Collinsella*, *Clostridium*, and *Ruminococcaceae*, correlate with the severity of RA [26]. This suggests a potential target for therapeutic interventions aimed at modulating the gut microbiota to mitigate the impact of rheumatic diseases.

Oral Microbiota and Its Role in the Treatment of Rheumatoid Arthritis

**Figure 1:**
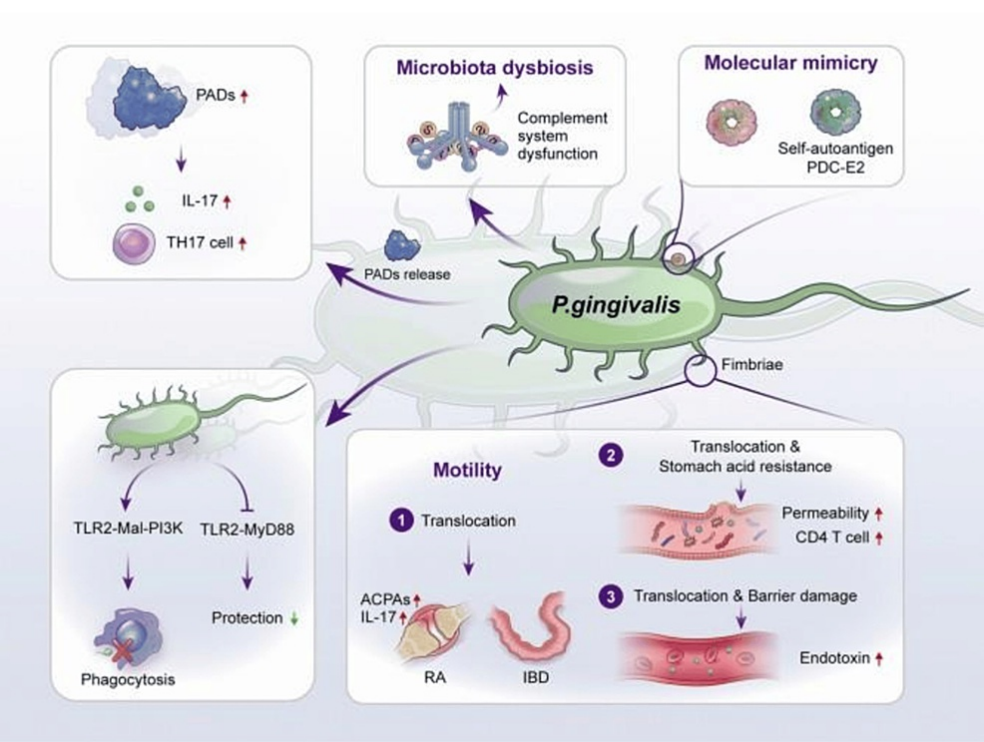
Graphical description of the possible mechanism of Gram-negative anaerobic bacillus Porphyromonas gingivalis for triggering autoimmune diseases Image credit: Reproduced with permission from Huang et al. [27]. ACPAs: Anti-citrullinated protein antibodies; PADs: Peptidylarginine deiminases; PDC-E2: Pyruvate dehydrogenase complex E2; IBD: Inflammatory bowel disease; RA: Rheumatoid arthritis.

**Figure 2:**
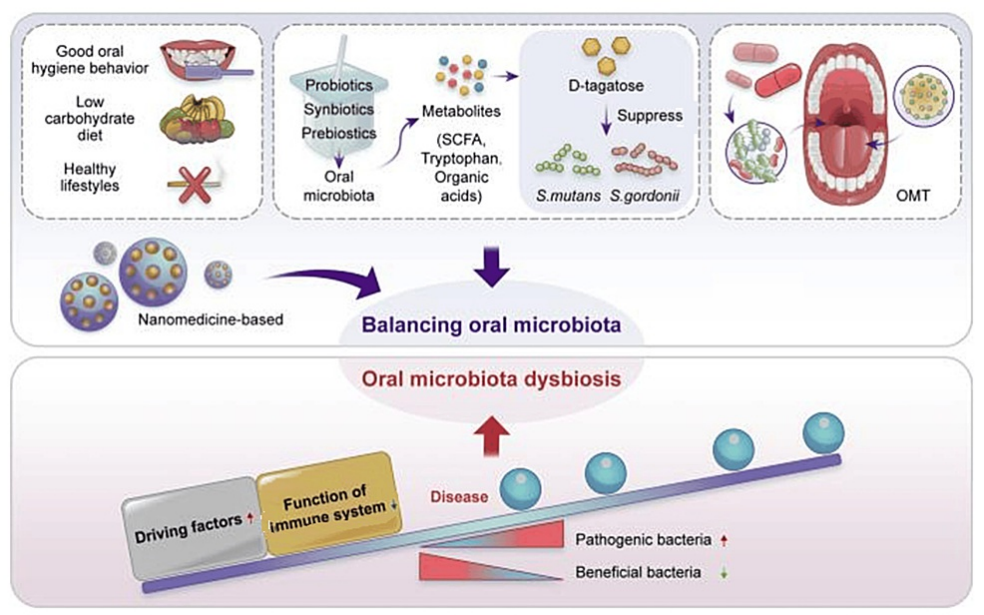
Treatment strategy for balancing oral microbiota to prevent autoimmune diseases Image credit: Reproduced with permission from Huang et al. [27]. SCFA: Short-chain fatty acids; OMT: Oral microbiota transplantation.

Research has demonstrated a close correlation between *P. gingivalis* and the occurrence and development of RA [27]. The molecular mechanism for this association is described in Figure 1. A higher prevalence of *P. gingivalis* in the tongue biofilms was observed in non-remission patients compared to remission patients [27]. The expression of *P. gingivalis* was significantly higher in patients with anticyclic citrullinated peptide-positive antibodies compared to controls [28]. Moentadj et al. found an abundance of *Streptococcus *species in the oral cavity of RA patients, and similarly, another case-control study reported a high prevalence of treponema in patients with Parkinson's disease and RA [29,30]. As oral microbiota has a significant role in dysbiosis resulting in the initiation and progression of autoimmune diseases, targeting this could be an effective strategy in the management of rheumatic diseases [27,28]. The risk of rheumatoid and autoimmune diseases can be reduced by maintaining the dynamic homeostasis between the oral microbiota and the host immune system. A healthy lifestyle, a low carbohydrate diet, and good oral hygiene can reduce the oral microbial load, and the use of prebiotics, probiotics, or synbiotics enhances the resilience of oral microflora and reverses the oral microbiota dysbiosis (Figure 2) [27].

Targeting Toll-Like and Nucleotide-Binding Oligomerization Domain-Like Receptors

Exploring potential therapeutic strategies to target toll-like receptors (TLRs) or nucleotide-binding oligomerization domain-like receptors (NLRs) emerges as a viable approach to alter the gut microbiota composition in RA patients [26]. These receptors play crucial roles in the immune response and could be manipulated to influence the microbiome in a way that is conducive to managing rheumatic diseases. This avenue recognizes the immunomodulatory potential of the gut microbiota and its connection to the pathophysiology of RA.

Modulating Antigenic Mimicry and Gut Permeability

Addressing antigenic mimicry, alterations in the permeability of intestinal mucosa, and control of the host immune system represent potential therapeutic strategies to alter the gut microbiota composition in RA patients [26]. This multifaceted approach recognizes the complex interactions between the gut microbiome and the host’s immune system, offering potential points of intervention to influence the course of rheumatic diseases.

Preventing Citrullination and Targeting T Helper Type 17-Mediated Inflammation

Enzymatic action to prevent citrullination of peptides by the gut microbiota and targeting T helper type 17-mediated mucosal inflammation could be specific therapeutic targets for RA [26]. These strategies acknowledge the role of microbiota-derived processes in contributing to the inflammatory milieu associated with rheumatic diseases. By addressing specific enzymatic actions and immune pathways, there is potential to modulate the disease course.

Impact on Gut Barrier Function and Immune Modulation

Exploring potential therapeutic strategies to target the impact of the gut microbiome on gut barrier function and the modulation of the immune system emerges as a comprehensive approach to managing rheumatic diseases [25]. This recognizes the broader influence of the microbiome, extending beyond specific microbial taxa, to impact the overall gut environment, immune responses, and the production of metabolites. However, it is essential to note that while these potential therapeutic strategies show promise, further studies, particularly in human populations, are imperative to validate their efficacy and safety [26].

Challenges to Harnessing the Microbiome in the Treatment of Rheumatic Diseases

Ethical challenges: Although many studies have focused on the role of the microbiome in the development of rheumatic diseases, the exact challenges involved in harnessing the microbiome for treatment remain unclear [5]. However, it is known that the complexity of the microbiome and its variability pose difficulties [5].

Social challenges: In fact, intestinal dysbiosis has been implicated in the development of distal synovial inflammation [5]. This can be seen in conditions like Whipple’s disease and arthritic syndrome triggered by jejunoileal bypass surgery. Additionally, gut-infecting or colonizing bacteria have been known to cause distal arthritis in various spondyloarthropathies [5].

Economic challenges: The microbiome composition can also be affected by host genetics and gender, with alterations in the intestinal microbiota composition leading to poorer outcomes for experimental murine models [5]. Moreover, exposure to the gut microbiota and downstream immune response is a challenge, as seen in the case of IL-1 receptor-antagonist knock-out mice, which develop a spontaneous autoimmune T-cell-mediated arthritis when raised under conventional cages, but not in GF animals [5].

Treatment modulation challenges: To overcome these challenges, numerous groups have been attempting to modulate bacterial composition or its byproducts to abrogate inflammatory responses in rheumatic diseases [5]. These methods include individual species or their metabolites and involve ecosystem-level interventions and single-target approaches [5].

Analytical and classification challenges: However, the bioinformatics pipelines and statistical tests used for microbiome analysis vary among studies [31]. Moreover, classifying bacteria into operational taxonomic units (OUT) using 97% homology is not always accurate, and the lack of functional studies to determine the mechanism through which gut microbiota might predispose to or protect from disease, combined with the fact that as little as 10% of taxa might be shared across a given population, makes it difficult to perform intra- and inter-studies comparison [31].

Technical challenges: Furthermore, there are technical challenges in terms of sample collection, storage, DNA extraction, and library preparation, including a selection of 16s primers and bacterial abundances that need to be analyzed using optimal and appropriate statistical tests like gene expression analysis [31].

Potential ethical implications involved with harnessing the microbiome in the treatment of rheumatic diseases

Advancements in Microbiome Research and Ethical Considerations

A better understanding of the microbiome’s role in rheumatic diseases is crucial [11]. Recent progress in microbiome research and tools has unveiled novel associations between gut microbiota and autoimmune diseases, offering potential insights for diagnosis, treatment, or prevention of conditions like cancer [32]. However, ethical considerations surround the medical use of the microbiome [8]. Concerns range from potential risks associated with the inadvertent handling of environmental toxins to ethical issues arising from research involving indigenous populations [33]. Informed consent is paramount before conducting any research in this domain [34]. Nanobiosensors utilizing living cells are employed in microbiome diagnostics, emphasizing the importance of technological advances [31]. The immense potential of harnessing the microbiome to alleviate spondylarthritis and rheumatoid arthritis burden is under exploration through empirical and experimental studies [35-37]. For future progress, research should focus on technological advancements to expedite discoveries [38]. Additionally, resolving molecular problems associated with microbiome-related diseases could pave the way for targeted microbiome therapeutics [39].

Addressing Ethical Implications and Future Considerations

Before implementing interventions like probiotics, it is crucial to ensure they are the last resort, considering traditional or alternative treatments to avoid desperate situations [40-43]. A science-based framework has been proposed to guide the ethical and environmentally safe use of probiotics, emphasizing steps such as environmental safety management, ethical considerations, and assessing potential environmental damage [40]. Ethical implications of microbiome management, particularly concerning the food chain, should be carefully considered [41-44]. Despite potential drawbacks, microbiome stewardship and scientific networks can facilitate the rapid development of probiotic applications [40]. Policies for production and safety should be crafted to meet regulatory standards, with pilot-scale experiments providing baseline evidence before widespread application [40]. However, the use of beneficial microbes is hindered by a lack of risk assessment and ethical frameworks, necessitating a careful balance between the risk of inaction and the need for rapid action [40]. Selecting and applying probiotics for wildlife involves science-informed exclusion of potential pathogens and consideration of ethical and environmental safety management [40,45]. Global debates with stakeholders, independent scientific entities, and community consultations aligned with international regulations should inform decisions [40,45].

Limitations

There are several limitations to our study findings, the most notable of which is that the findings come from non-randomized clinical trials. The lack of statistical tests is another limitation of this study. Most microbiome studies showed inconsistent results due to variation between the individuals in these studies, which can be minimized through randomized controlled clinical trials. Future studies can provide useful results by collecting intestinal and oral samples in RA patients, thereby allowing researchers to analyze the simultaneous effect of oral-gut microbiomes in the same individuals, which is missing in most current studies. Therefore, more research is required on the combined therapeutic targets and optimal microbiological environment.

## Conclusions

In summary, machine learning analytics of metagenomic data offers insights into the microbiome's role in rheumatic diseases, highlighting distinctive features and potential diagnostic markers. New therapeutic strategies, including combination therapies based on the "treat-to-target" approach, are emerging, demonstrating improved responses. Ethical considerations are crucial in implementing interventions like probiotics, emphasizing the need for a science-based framework to guide their ethical and environmentally safe use. The microbiome's potential in treating rheumatic diseases is evident from studies linking specific bacteria to protective or deleterious roles and the impact of dysbiosis on synovial inflammation. Probiotics, dietary modifications, and medications show promise in modulating the microbiome for therapeutic benefits in rheumatic diseases. While challenges like the difficulty of controlling the environment persist, the microbiome remains a promising avenue for personalized therapeutic strategies. Ongoing research aims to deepen our understanding of microbiome mechanisms and develop more effective treatments.
